# Ovariotomy under Difficulties

**Published:** 1874-05

**Authors:** 


### Ovariotomy under Difficulties.

Dr. S. G. Stevens writes to the *Medical Times and Gazette* of March 7, 1874, from Rio Bueno, Valdivia, Chili, an account of an operation for the removal of an ovarian tumor performed by himself in the backwoods of South America. The operator showed great skill in the use of the few instruments he could command, and of such materials as he could find at hand.

The patient was an Indian woman, about 38 or 40 years of age, married, who had borne nine children, and who lived near the Lake of Ranco, in the great forests at the foot of the Cordillera. Dr. Stevens first saw her in January, 1871, having passed her hut to ask for lodgings for the night. She was lying in one corner of the hut, on her bed of sheepskins, evidently suffering greatly. She said that about five years previously, she had had a quarrel with another woman, who had suddenly disappeared from the neighborhood, and that ever since her belly had been swelling; the other woman had bewitched her and that unless she could find her—for the Indians believe that the person who has caused them the evil can cure them—or some remedy, she would go on swelling, burst and die. She was suffering from great dyspnœa. Her countenance was livid, and the abdomen, of enormous size, fluctuated distinctly. Dr. Stevens told her he thought he could take out the evil, and her husband consented to the trial. The intention was to make an exploratory incision, and extirpate the tumor, if possible; if not, to tap and close up the wound. Dr. Stevens set about preparing for the operation, while a messenger was sent four days' journey to obtain chloroform. The instruments were a trocar, made from a piece of bamboo, about ten inches long, hollowed out, and sharpened to a point at one end, and at the other connected to a piece of India-rubber tubing from an enema syringe; the instruments from a "Charrière" pocket-case, and a pair of craniotomy forceps. The assistants were a Catholic Missionary, two Indians, and a half-blood. The ligature was of raw hide, with two pieces of wood fastened at the ends, in order that more power could be used in pulling it tight; and at the time of using it was to be dipped in warm neats-foot oil.

Jan. 25th.—After the hut had been heated, and the patient brought under the influence of chloroform the operation was begun. An incision four inches long was made in the linea alba down to the peritoneum. The wound having been dried with cotton wool, a small incision was made in the peritoneum, when the fluid gushed out like a fountain into the face of the operator. On enlarging the incision the tumor presented. It appeared solid at first, but a point of fluctuation was discovered. On the introduction of the trocar, the fluid ran well at first, but gradually became thicker, and after the tubing had been disconnected it soon ceased to flow from the bamboo tube. On enlarging the opening, another cyst was found which was emptied in the same way as the first, and on introducing the hand behind the tumor, it was found free in every part. The craniotomy forceps were applied, and the wound being extended a little at its upper part, the tumor came out upon the board, which was in readiness for its reception, and behind it a rush of ascitic fluid. The pedicle was rather long, but flat; the raw-hide ligature was applied to it, and tightened by means of the two pieces of wood, pulled by the two assistants on each side of the body until it was almost buried in the parts, and then made fast with two lasso-knots, the ends cut off, and the whole dropped into the cavity. The cavity was mopped out with cotton wool, and the wound closed with fine, iron-wire sutures, and a superficial, continuous suture of silk. Water dressing was next applied, and a warmed flannel roller passed twice round the body. Consciousness returned before the patient could be removed from the table, owing to the priest not attending to the chloroform, being too much occupied with, and astonished at, the operator's movements. Great exhaustion followed, but, under the use of stimulents, the patient rallied.

Dr. Stevens remained in the neighborhood twelve days, during which time the patient for the most part did well. The first pair of sutures were removed Jan. 28th, and day by day one or more was removed, until the ninth or middle one was taken out.

February 26th, the wound had entirely healed, but she complained of pain in the left side near the pubes. Rest, good diet, and care as to the state of the bowels were ordered.

March 15th—She could stand at the door of the hut to receive Dr. Stevens. Had no pain but was still very weak.

June 28th.—She came from the lake to the village of Rio Bueno to see her physician—a days' journey through the forest. The menses had returned, and she was quite well.

The patient continued well until Dec., 1872—nearly two years—when she, with her husband, went to the funeral of a relative, drank and became intoxicated. On passing a river, her horse stumbled and fell, and she was drowned.

The tumor consisted of five large cysts, and many smaller ones. Weight, without the fluid, thirteen pounds and a half.

Dr. Stevens had never seen the operation of ovariotomy, nor read any special work on the subject. He had nothing to direct him but the short account given in Tanner's "Practice of Medicine." —*Boston Med Journal.*

